# A review of probiotics in the treatment of autism spectrum disorders: Perspectives from the gut–brain axis

**DOI:** 10.3389/fmicb.2023.1123462

**Published:** 2023-03-16

**Authors:** Pengya Feng, Shuai Zhao, Yangyang Zhang, Enyao Li

**Affiliations:** ^1^Department of Children Rehabilitation, Key Laboratory of Rehabilitation Medicine in Henan, The Fifth Affiliated Hospital of Zhengzhou University, Zhengzhou, Henan, China; ^2^Key Laboratory of Helicobacter pylori, Microbiota and Gastrointestinal Cancer of Henan Province, Marshall Medical Research Center, Fifth Affiliated Hospital of Zhengzhou University, Zhengzhou, China; ^3^College of Bioengineering, Henan University of Technology, Zhengzhou, China

**Keywords:** autism spectrum disorders, probiotics, gut microbiota, gut–brain axis, gastrointestinal abnormalities

## Abstract

Autism spectrum disorders (ASD) are a class of neurodevelopmental conditions with a large societal impact. Despite existing evidence suggesting a link between ASD pathogenesis and gut–brain axis dysregulation, there is no systematic review of the treatment of probiotics on ASD and its associated gastrointestinal abnormalities based on the gut–brain axis. Therefore, we performed an analysis for ASD based on preclinical and clinical research to give a comprehensive synthesis of published evidence of a potential mechanism for ASD. On the one hand, this review aims to elucidate the link between gastrointestinal abnormalities and ASD. Accordingly, we discuss gut microbiota dysbiosis regarding gut–brain axis dysfunction. On the other hand, this review suggests that probiotic administration to regulate the gut–brain axis might improve gastrointestinal symptoms, restore ASD-related behavioral symptoms, restore gut microbiota composition, reduce inflammation, and restore intestinal barrier function in human and animal models. This review suggests that targeting the microbiota through agents such as probiotics may represent an approach for treating subsets of individuals with ASD.

## 1. Introduction

Autism spectrum disorders (ASD) are severe neurodevelopmental disorders that first manifest in newborns and young children (Li and Zhou, 2016). It is marked by deficiencies in social and linguistic skills as well as repetitive behavior patterns (American Psychiatric Association, 2013). According to the Global Burden of Diseases, Injuries, and Risk Factors Study from 2016, 62.2 million individuals worldwide are considered to have ASD (Vos et al., 2017). In addition, its incidence appears to increase over time (Li et al., 2022). Therefore, research on ASD and development of clinical treatment for it are increasingly important.

Numerous comorbidities including epilepsy, anxiety, depression, Tourette syndrome, tic disorders (Howes et al., 2018), gastrointestinal (GI) problems (Chaidez et al., 2014), and intellectual disability are linked to ASD (Autism and Developmental Disabilities Monitoring Network Surveillance Year 2008 Principal Investigators, 2008). Among them, GI problems, such as abdominal pain, constipation, and diarrhea, are the common comorbidities affecting 9 to >70% of children with ASD (Frye and Rossignol, 2016). These GI disorders can be difficult to treat since they are often resistant to standard therapy (Frye and Rossignol, 2016). These GI problems are possibly linked to gut bacteria. The gut–brain axis, which describes the reciprocal interaction between the central nervous system (CNS) and the trillions of microorganisms that reside in the gut, is a potential pathway by which changes in gut microbiota may affect brain functions and development (Wang and Wang, 2016). Thus, the composition and function of gut microbiota can be important for ASD treatment. In this review, we focus on the applicable mechanisms whereby observe how probiotics can be used to treat GI symptoms and central symptoms of ASD through the gut–brain axis.

## 2. Gastrointestinal abnormalities in ASD

**Figure 1:**
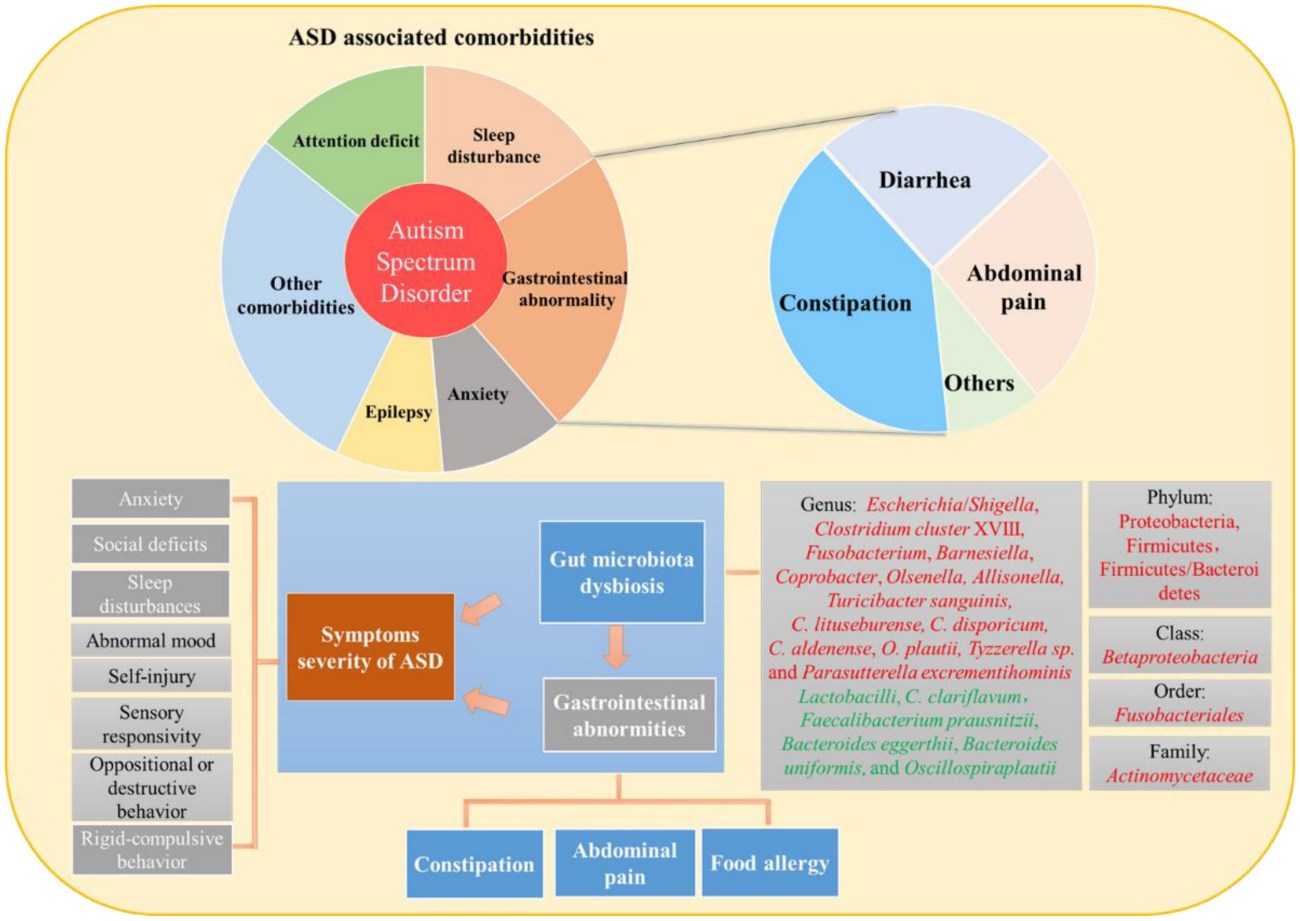
Interrelationship between gut microbiota dysbiosis, gastrointestinal abnormities, and symptoms severity of ASD.

Numerous studies have suggested that patients with ASD often suffer from GI abnormalities; however, the pathogenesis of ASD-related GI problems is not yet fully understood. A recent study has reported two hypotheses for GI abnormalities in ASD (Navarro et al., 2016). One study hypothesized that GI abnormalities may be a manifestation of an underlying inflammatory process, which may be pathophysiologically related to abnormal microbiota. For example, gut microbiota dysbiosis contributes to the pathophysiology of many GI conditions such as inflammatory bowel disease and functional GI disease (Cammarota et al., 2014). The second hypothesis, the functional bowel disease hypothesis, considers that GI abnormalities in ASD may be simply a reflection of sensory over-responsivity to abdominal signals. Gut microbiota dysbiosis, GI abnormities, and ASD symptoms severity show strong relationships (Figure 1). Gastrointestinal abnormalities unrelated to any underlying anatomical or metabolic abnormalities often accompany ASD in humans (Gorrindo et al., 2012). According to a meta-analysis, children with ASD were four times more likely to experience general GI issues, three times more likely to experience constipation or diarrhea, and two times as likely to experience stomach pain (McElhanon et al., 2014). In most cases, the underlying cause for these symptoms was usually recognized as GI abnormalities.

### 2.1. Gastrointestinal abnormalities (abdominal pain and constipation) correlate with symptom severity of ASD in humans

The diagnosis of GI abnormalities is typically indicated by certain behavioral complications (Maenner et al., 2012). A previous study reported that GI abnormalities (assessed by the 6-GSI) significantly correlate with symptom severity in ASD (assessed by the autism treatment evaluation checklist) (Adams et al., 2011). Furthermore, constipation is the most common GI symptom observed in autistic children (Srikantha and Mohajeri, 2019). Moreover, the presence and intensity of abdominal pain have been directly associated with the severity of ASD core symptoms (Ding et al., 2017). Such findings suggested a gut–brain axis-mediated relationship between GI anomalies in ASD and behavioral output (Hsiao, 2014). In addition, GI abnormalities have shown a correlation with other ASD comorbidities, such as sleep difficulties, abnormal mood, and social deficits. In comparison with ASD patients without GI symptoms, it has been discovered that GI comorbidity in patients with ASD was associated with increased sleep issues, abnormal mood, argumentative, oppositional, defiant, or destructive behavior, anxiety, sensory responsiveness, rigid compulsive behaviors, self-injury, aggression, lack of expressive language, and social impairment (Nikolov et al., 2009).

### 2.2. Gut microbiota dysbiosis is associated with ASD-related GI symptoms (constipation, food allergy, and abdominal pain)

Increasing evidence has shown ASD children with constipation have higher relative abundances of *Escherichia*/*Shigella* and *Clostridium cluster* XVIII (Strati et al., 2017), the order Fusobacteriales, the family Actinomycetaceae, and the genera *Fusobacterium*, *Barnesiella*, *Coprobacter*, *Olsenella,* and *Allisonella* (Liu et al., 2019), as well as lower *Faecalibacterium prausnitzii*, *Bacteroides eggerthii*, *Bacteroides uniformis*, *Oscillospira plautii*, and *Clostridium* (*C.*) *clariflavum* amount (Luna et al., 2017). Moreover, the lower abundance of *Lactobacilli* (Iovene et al., 2017) could be related to constipation in patients with ASD because its depletion was connected with chronic constipation in non-ASD children (Kushak et al., 2017). Patients with ASD who also had allergies had higher relative abundances of the phylum Proteobacteria in their stools, previously linked to autoimmune diseases (Kong et al., 2019). In addition, cecal *Betaproteobacteria*, ileal and cecal Firmicutes, and the Firmicutes/Bacteroidetes ratio appear to increase in association with food allergies (Williams et al., 2011). It was found that Firmicutes/Bacteroidetes ratio is negatively correlated with allergy/immune function in feces in ASD children (Kong et al., 2019). *Turicibacter sanguinis*, *C. lituseburense*, *C. disporicum*, *C. aldenense,* and *O. plautii* levels were higher in ASD children who experienced GI discomfort. Some bacteria may be associated with >1 GI symptoms, for instance, *C. aldenense* and *O. plautii* have been also identified in ASD patients with constipation (Luna et al., 2017). Interestingly, some ASD children have extremely high levels of certain bacteria that are positively connected with GI symptoms (i.e., *Turicibacter sanguinis*) (Kang et al., 2013). More recently, Parracho et al. (2005) demonstrated that ASD children have higher fecal content of the *C. histolyticum* group-known toxin producers (Hatheway, 1990) than healthy unrelated controls but not than healthy siblings. In addition, high levels of *Clostridium* species were substantially related to GI issues in patients with ASD, including those with and without GI symptoms.

## 3. Impaired gut–brain axis in ASD

The hypothalamic–pituitary–adrenal axis, the vagus nerve, the sympathetic and parasympathetic nervous systems with the enteric nervous system, as well as the neuroendocrine and neuroimmune systems are considered to form the gut–brain axis, a biochemical bidirectional signaling pathway between the gut and the brain (Dinan and Cryan, 2015). A growing number of studies has demonstrated a role for it in the etiology of ASD (Li et al., 2017). Brain function was influenced by the gut microbiota *via* neuroendocrine, neuroimmune, and autonomic nervous systems (Mayer, 2011).

### 3.1. Gut microbiota dysbiosis leads to immune system dysregulation

The gut microbiota dysbiosis in autism usually results in immune system disorders (Doenyas, 2018). Interleukin-1 (IL-1), interleukin-6 (IL-6), interferon (INF), and tumor necrosis factor (TNF) are chemokines and cytokines that are released by the active immune system which may cross the blood–brain barrier. These mediators attach to brain endothelial cells, triggering immunological reactions (de Theije et al., 2011). A previous study found significantly higher IL-1, IL-6, and IL-8 plasma levels in the ASD group than in the typical development controls (Ashwood et al., 2011). In addition, the immune system is concentrated in and around the gut mucosa, where around 80% of it is located (Critchfield et al., 2011).

### 3.2. Gut microbiota metabolism dysbiosis contributes to ASD

**Figure 2:**
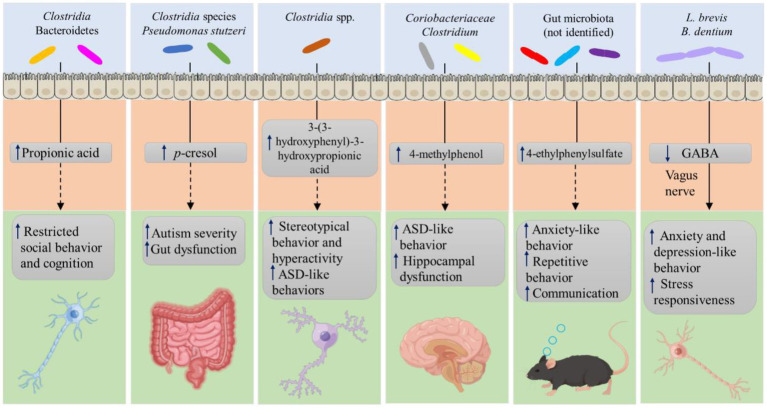
Gut microbiota-derived metabolites contribute to ASD.

Patients with ASD have variable bacterial diversity. According to several studies, they have significantly decreased species diversity and richness (Carissimi et al., 2019; Ma et al., 2019), whereas other studies found the opposite (Finegold et al., 2010; De Angelis et al., 2013). The gut microbiota affects brain physiology through its differential metabolites (Figure 2). Patients with ASD have been shown to have an increase in the level of metabolites including SCFAs, p-cresol, and ammonia, in serum, urine, and fecal samples, which can cause behavioral symptoms and symptoms resembling autism by the vagal pathway (Forsythe et al., 2014). Among these, SCFAs, including acetic acid, propionic acid, butyrate, isobutyric acid, valeric acid, and isovaleric acid, have been considered the major signaling metabolites, which play a critical role in regulating catecholamine production throughout life and in preserving the neurotransmitter phenotype after birth, and have been shown to be important in ASD (Wang et al., 2012). However, some studies found lower levels of these SCFAs, except for propionic and acetic acid, in children with ASD. *Clostridium* and Bacteroidetes can produce propionic acid, which can penetrate the blood–brain barrier and cause autism-like behaviors, such as impaired and restricted social, behavior, and cognition, by modulating 5-Hydroxytryptamine (5-HT) and dopamine (DA) in the brain (Thomas et al., 2012). In addition, propionic acid decreases the levels of intracellular antioxidants such as GSH and superoxide dismutase and the production of pro-inflammatory cytokines (Wajner et al., 2004). Increased oxidative stress and inflammation are known to play an important role in the pathogenesis of ASD (Bjørklund et al., 2020). Children with autism have been shown to have higher levels of the microbial metabolite p-cresol and its conjugate p-cresyl sulfate in their urine samples. *Clostridia* species and *Pseudomonas stutzeri* strains may explain the high p-cresol levels (Altieri et al., 2011). In addition, increasing serum levels of 4-methylphenol, a minor aromatic metabolite generated by gut bacteria, causes ASD-like behavior and hippocampus impairment (Liu et al., 2022). Moreover, ASD patients’ urine contains higher levels of 3-(3-hydroxyphenyl)-3-hydroxypropionic acid, a phenylalanine metabolite generated by *Clostridia* spp., which may be responsible for the depletion of catecholamines that worsens stereotyped behavior and hyperactivity (Shaw, 2010). In addition, it has been connected to ASD-like behaviors in mouse models. Particularly, offspring of dams treated with the inflammatory molecule poly (I: C) show changes in gut microbiota composition and dysregulation of metabolite concentrations in the serum, including elevated levels of the microbial metabolite 4-ethylphenylsulfate, which led to anxiety-like behavior in mice otherwise untreated (Hsiao et al., 2013). In addition, 5-aminovaleric acid and taurine levels were reduced in recipient mice microbiota from persons with ASD, and both these metabolites can act as aminobutyric acid (GABA) receptor agonists (Sharon et al., 2019). In fact, in the BTBR T + Itpr3tf/J mouse model of ASD, treatment with these two metabolites was effective in reducing repetitive behaviors and improving sociability (Sharon et al., 2019). Tryptophan’s metabolite, indole, serves as a precursor for crucial chemicals including 5-HT and DA (De Angelis et al., 2013) and is able to be synthesized by *Alistipes* that are higher in individuals with anxiety and depression (Zhang et al., 2015), ultimately disrupting the serotonergic balance in the body. Therefore, an aberrant increase or decrease in gut microbiota-derived metabolites can worsen the symptoms of ASD.

## 4. Probiotics improve ASD by regulating gut–brain axis

**Figure 3:**
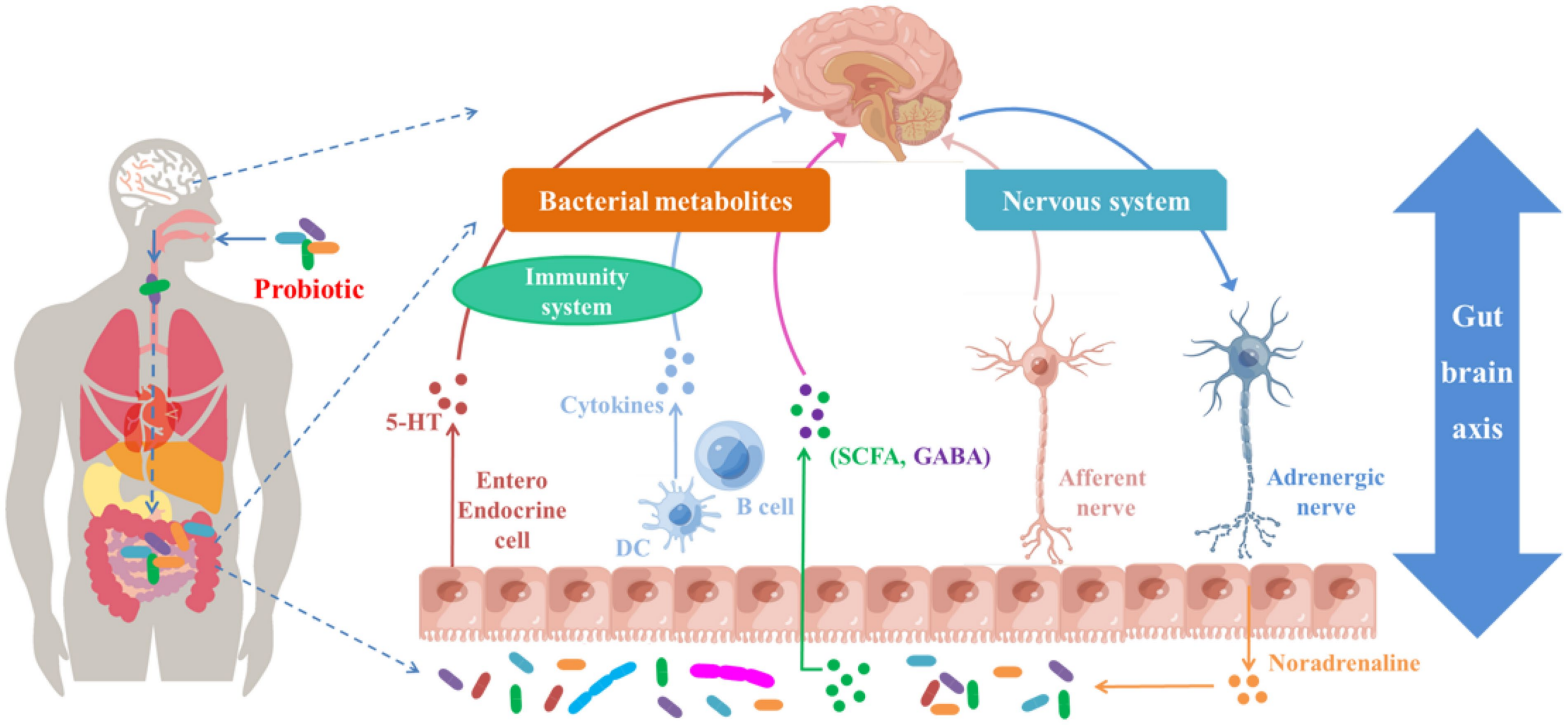
Potential ASD treatment responses triggered by probiotics and their metabolites through gut–brain axis.

Hence, modulating the microbiota–gut–brain axis with probiotics could be an effective strategy for ASD improvement (Figure 3) and may alleviate GI dysfunction. Several trials have used probiotics to effectively treat GI disorders such as traveler’s diarrhea (McFarland, 2007) and irritable bowel syndrome (Saggioro, 2004). We consider the clinical trials using probiotics in children with ASD are justified based on the similar symptoms, the presence of toxin-producing *Clostridium* species in ASD persons, the evidence that the achievements in treating irritable bowel syndrome, and the suppression of *Clostridium* with probiotics. Recently, probiotic therapy has been described as an additional and alternative treatment for ASD (Tas, 2018; Cekici and Sanlier, 2019). Children with ASD aged 5–9 years who received probiotic supplements for 3 months showed improvements in their GI microbiota, GI symptoms, and the severity of their ASD symptoms, behaviors, and functioning (Shaaban et al., 2018). Similarly, a multi-strain combination of 10 probiotics administered for 4 weeks to a 12-year-old child with ASD decreased GI symptoms and improved ASD core symptoms (Grossi et al., 2016).

### 4.1. Clinical evidence that probiotics regulate gut–brain axis to alleviate ASD symptoms

There is evidence that probiotic supplementation improved the behavior of ASD children through the gut–brain axis (Table 1). The effect of probiotics on psychological conditions such as depression and anxiety is relatively well known (Ng et al., 2018). Children with autism who received vancomycin orally and probiotic *Bifidobacterium* supplements had significantly higher urine levels of 3-(3-hydroxyphenyl)-3-hydroxyproionic acid, 3-hydroxyphenylacetic acid, and 3-hydroxyhippuric acid (Xiong et al., 2016). The first metabolite can cause autistic symptoms by lowering catecholamine levels in the brain (Li and Zhou, 2016). Thus, the decreased levels of those metabolites may be responsible for improved eye contact and less constipation in children with autism (Xiong et al., 2016). A recent study found that probiotics could improve the brain activity of preschoolers with ASD. This was demonstrated by a reduction in frontopolar region power in the beta and gamma bands, a decrease in frontopolar region coherence in the same bands, and a change in frontal asymmetry using electroencephalography (EEG) (Billeci et al., 2022). Beta waves are connected to physiological activity, focus, analytical thought, and states of specific mental commitment or motor activities (Tallon-Baudry, 2003), whereas gamma waves are associated with working memory tasks and several early sensory reactions. When compared to typically developing persons, ASD brains’ resting EEGs frequently show enhanced beta and gamma spectral band activity (Nicotera et al., 2019). Abnormal GABAergic tone in the growth of plasticity and brain function is expected to be involved in the regulation of the EEG frequency bands, which may be partially responsible for the atypical increase in high-frequency bands in ASD (Baumgarten et al., 2016). One of the main features of the neurophysiology of ASD is an altered GABA (the CNS primary inhibitory neurotransmitter) pattern. Atypical brain excitation/inhibition balance, altered neuronal signaling, information processing, and responsive behavior, in particular, may be caused by the deficient inhibitory GABAergic signaling that characterizes patients with ASD (Foss-Feig et al., 2017). After probiotic supplementation, the brain activity of ASD children (showing an improvement in excitatory/inhibitory imbalance) suggested that probiotics can promote a change in brain activity in ASD children toward that of controls. Moreover, probiotic administration was found to promote a shift in brain connections toward a more typical pattern with respect to coherence and asymmetry. Importantly, probiotics could significantly improve the brain function of animals with ASD. For example, immunohistochemical analysis of brain tissues showed that *B*. *longum* CCFM1077 could ameliorate microglia activities in the cerebellum of autistic rats, as evidenced by the decreased *IBA-1* protein expression (Kong et al., 2022). Furthermore, oral probiotics (containing *B. bifidum*, *B. infantis,* and *L*. *helveticus*) could inhibit MIA-induced decrease in PV^+^ neuron numbers in the PFC in adult offspring (Wang et al., 2019). In addition, treatment with *Lactobacillus* strains reversed the VPA-induced apoptosis and degeneration in the cerebellum (Sunand et al., 2020). All the aforementioned studies suggested that the recovery of brain function after probiotics treatment provides important evidence for the connection between the gut and the brain.

**Table 1:** Effect of probiotic supplementation on the health status of individuals with ASD.

Probiotics	Species	Dose and duration	Effects	References
*L*. *plantarum* WCSF1	Children with ASD, 4–16 years old	4.5 × 10^10^ CFU per capsule per day for 3 weeks during the 12 weeks study duration	Improve behavioral scores and the stool consistency, increase Enterococci and Lactobacilli group, decreased *Clostridium* cluster XIVa	Parracho et al. (2010)
Any type of probiotic	Children with ASD, 2.5–18 years old	Daily usage (33%)	Lower levels of total SCFAs; Marginally elevate the level of *Lactobacillus*	Adams et al. (2011)
*L*. *acidophilus* Rosell-11	Autistic children, 4–10 years old	5 × 10^9^ CFU per gram twice a day for 2 months	Decrease D-arabinitol and D-arabinitol/L-arabinitol ration in urine	Kałużna-Czaplińska and Błaszczyk (2012)
*L*. *delbruecki*, *B*. *longum*		10^10^ CFU per capsule,3 times a day for 6 months	Decrease the ATEC score, improve speech/language communication, sociability, sensory cognitive awareness, and health/physical behavior	West et al. (2013)
3 *Lactobacillus* strains, 2 *Bifidobacterium* strains, and a *Streptococcus* strain (60:25:15 ratio)	Children with ASD, 2–9 years old	3 capsules per day (1 capsule thrice a day) for 4 months	Normalize Bacteroidetes/Fircumutes ratio, increase *Bifidobacterium*, and reduce *Desulfovibrio* spp. and TNF-α level in feces	Tomova et al. (2015)
*L. delbrueckii* subsp. *Bulgaricus*, *L*. *acidophilus*, *B*. *breve*, *B*. *longum*, *B*. *infantis*, *L*. *paracasei*, *L*. *plantarum*, *S*. *thermophiles*	Children with ASD, 12 years old	5 months of treatment period (4 weeks of initial treatment +4 months of follow up treatment);10 months of follow up period	Improve autistic core symptoms and abdominal symptoms	Grossi et al. (2016)
*Saccharomyces boulardii*	A 16-year-old boy with Autism	3 × 10^9^ CFU per capsule, initiated at 6 capsules daily (2 at breakfast, 2 at lunch, 1 at dinner, and 1 at bedtime), 12 capsules daily after 1 weeks, and 24 capsules after 3 months	Reduce obsessive compulsive disorder and self-injurious behavior	Kobliner et al. (2018)
*B*. *longum*, *L*. *rhamnosus*, *L*. *acidophilus*	Autistic children, age from 5–9 years old	1 × 10^8^ CFU per gram, 5 g per day for 3 months	Decrease severity of the ASD and GI symptoms; Increase abundances of Bifidobacteria and Lactobacillus	Shaaban et al. (2018)
*L*. *rhamnosus*, *L*. *paracasei* and *B*. *longum*	Autistic children aged between 9–12 years old	2 × 10^10^ CFU, once daily for 6 weeks	Improve autistic symptoms (assessed by ATEC)	Tharawadeephimuk et al. (2019)
6 bacteria (the strain was not shown)	Children with ASD, age from 3–8 years old	Each bacteria was 1 × 10^9^ CFU/gram, 6 g per day, in combination with applied behavior analysis training for 4 weeks.	Alleviate the autism symptom (assessed by ATEC scores); Improve the GI symptom (assessed by a GI questionnaire)	Niu et al. (2019)
*S*. *thermophilus*, *B*. *breve*, *B*. *longum*, *B*. *infantis*, *L*. *acidophilus*, *L*. *plantarum*, *L*. *paracasei*, *L*. *delbrueckii* subsp. bulgaricus	Children with ASD, age range from 18–72 months	4.5 × 10^11^ bacteria each packet, 2 packets/day in the first month and 1packet/day in the following 5 months	Decline the ADOS scores in ASD children without GI symptoms; Improve GI symptoms, adaptive functioning, and sensory profiles in ASD children with GI symptoms;	Santocchi et al. (2020)
*L*. *plantarum* PS128	Autistic children and adolescents aged 45–127 months	3 × 10^10^ CFUs and 6 × 10^10^ CFUs of the probiotic if children weight was less than 30 kg and a higher weight, respectively.	Improve the Clinical Global Impression (CGI) scores	Mensi et al. (2021)
*L*. *plantarum* PS128	Individuals with ASD aged 3–20 years	Combination therapy of daily 2 capsules (6 × 10^10^ CFUs) for 28 weeks and oxytocin starting on week 16	Improve social and behavioral measurements, the ABC total score, ABC stereotyped behavior sub-score, and SRS cognition sub-score in a trend; Significantly improve Clinical Global Impression; enrich beneficial bacteria (*Blautia*, *Barnesiella*, *Christensenellaceae*R7, and *Ruminococcaceae* UCG-002) in the gut; decrease IL-1β in serum	Kong et al. (2021)
*S*. *thermophilus*, B. breve, *B*. *longum*, *B*. *infantis*, L. acidophilus, *L*. *plantarum*, *L*. *paracasei*, and *L*. *delbrueckii* subsp. Bulgaricus	Children aged 18–72 months diagnosed with ASD	A commercial probiotics formulation (the number of bacteria was not shown)	Decrease the power in frontopolar regions in β and γ bands, increase coherence in the same bands, and shift the frontal asymmetry	Billeci et al. (2022)
*Bifidobacterium* spp. and *Lactobacillus* spp.	Children with ASD aged 2–5 years	10^8^ bacteria/g, 10 grams daily for 3 months	Significantly increase *Bifidobacterium* spp. and *Lactobacillus* spp. in the stool; improve autism scale, sleep disturbances, communication to speak, social networking, and hyperactivity; reducing GI symptoms	Meguid et al. (2022)

### 4.2. Preclinical evidence that probiotics regulate gut–brain axis to alleviate autism

There is no clear explanation for the regulatory effects of probiotic supplementation on the gut–brain axis in humans, but there are numerous preclinical studies in animal models of ASD (Table 2). Probiotics have been shown to prevent *Candida* from colonizing the stomach (Romeo et al., 2011), and *Bifidobacterium* (*B*.) *longum* BB536 could modulate *Clostridium* (decreased the harmful *C. perfringens* and increased *Clostridium* cluster IV) populations and rescue social impairment in a rodent model of autism induced by PPA (Abuaish et al., 2021). Some *Clostridium* species generate p-cresol, which has been suggested as a potential urine biomarker for autism (Persico and Napolioni, 2013). Moreover, *Lactobacillus* (*L.*) *plantarum* ST-III could ameliorate the social deficits, self-grooming, and freezing times and increase the abundance of the beneficial *Lachnospiraceae* and decrease that of *Alistipes* in a mouse model of ASD (offspring of pregnant mice exposure to triclosan) (Guo et al., 2022). The gut microbiota contains several members of the *Lachnospiraceae* family, which has beneficial effects on human health (David et al., 2014), as they can increase the synthesis of the SCFAs acetate and butyrate (Byndloss et al., 2017) as well as boost the conversion of primary to secondary bile acids and reduce the generation of pro-inflammatory cytokines, being also crucial in supplying energy to the host (Smith et al., 2013). Tryptophan is transformed into indoles by *Alistipes*, which ultimately throws off the body’s serotonergic equilibrium. A previous study found a higher presence of *Alistipes* in depressed and anxious individuals (Zhang et al., 2015). Treatment with *L. helveticus* CCFM1076 significantly reduced *Turicibacter* abundance in the gut and increased butyric acid levels in the cecum contents of valproic acid (VPA)-treated rats (Kong et al., 2021). In the BTBR mouse model of autism, probiotic *L. rhamnosus* therapy favorably influences the microbiota–gut–brain axis favorably (Pochakom et al., 2022), as indicated by a reduction in behavioral deficits in social novelty preference, increased microbial richness, phylogenetic diversity, presence of potential anti-inflammatory (*Anaeroplasma* and *Christensenellaceae*) and butyrate-producing taxa (*Acetatifactor*, *Lachnospiraceae*, and *Butyricicoccus*), and elevation of 5-aminovaleric acid and choline in serum and in the prefrontal cortex (PFC), respectively. Moreover, a mixture of probiotics VSL#3 significantly improved sociability, social interaction, anxiety-liked behavior, and behavioral despair, while restoring the Bacteroidetes/Firmicutes ratio induced by prenatal VPA exposure (Adıgüzel et al., 2022).

**Table 2:** Effect of probiotic supplementation on the health status of animal models with ASD.

Probiotics	Species	Dose and duration	Effects	References
*L. rhamnosus* JB-1	Adult male BALB/c mice	1 × 10^9^ CFU of bacteria given orally every day for 28 days	Affect brain function through the vagus nerve	Bravo et al. (2011)
*Bacteroides fragilis* NCTC 9343	Offspring of pregnant C57BL/6 N mice injected i.p. on E12.5 with 20 mg/kg viral mimic poly(I:C)	10^10^ CFU in sugar-free applesauce over standard food pellets every other day for 6 days at weaning	Restore intestinal permeability, partly improve gut microbiota imbalance, improve communication, repetition, sensorimotor and anxiety-like behavioral abnormalities	Hsiao et al. (2013)
*L*. *reuteri* MM4-1A	Shank3 mutant mice	10^9^ bacteria reconstituted in a volume of 200 μL of PBS, twice a week for 3 weeks at 8 weeks of age	Attenuate unsocial behavior, decrease repetitive behaviors, and affect GABA receptor gene and protein levels in multiple brain regions	Tabouy et al. (2018)
*L*. *reuteri* MM4-1A	Offspring of C57Bl6/J mice access to HFD	10^8^ bacteria reconstituted in drinking water, access to water *ad libitum* for 4 weeks	Increase the oxytocin level of the hypothalamus and stimulate neurons in the ventral tegmental area of the midbrain	Buffington et al. (2016)
*B. bifidum*, *B. infantis* and *L. helveticus*	Offspring of pregnant C57BL/6 J mice injected i.p. on E12.5 with 20 mg/kg viral mimic poly(I:C)	1.9 × 10^8^ CFU/g *Bifidobacteria* and 6.4 × 10^9^ CFU/g *Lactobacillus* reconstituted in drinking water at concentration of 1.5 g/100 mL, access to water from embryonic day 0.5 to postnatal day 21	Restore MIA-induced weight loss in dams, social deficits, repetitive and stereotyped behaviors, depression-like behaviors, and anxiety-like behaviors in adult offspring; parvalbumin positive neuron loss; the decrease in levels of GABA in the PFC of adult offspring, and the decrease in proinflammatory cytokines (IL-6 and IL-17a) in both the maternal serum and fetal brain	Wang et al. (2019)
*L. plantarum*, *L. casei*, *L. acidophilus*, and *L. bulgaricus*	Offspring of the pregnant rats induced by VPA at a dose of 400 mg/kg, i.p. on an embryonic day 12	1 × 10^9^ CFU/mL of probiotics given orally every day for 42 days	Significantly attenuate the behavioral anomalies; Decrease the 5-HT, increase BDNF, IL-6, and TNF-α levels in blood and brain; Reverse the VPA-induced apoptosis and degeneration in the cerebellum	Sunand et al. (2020)
*L. helveticus* CCFM1076	Male offspring of pregnant Wistar rats injected i.p. on E12.5 with 500 mg/kg VPA	10^9^ CFU/mL bacteria daily gavage at age from 4 to 8 weeks	Improve social interaction, cognitive ability, and repetitive stereotyped behavior significantly; Up-regulate5-HT, L-Trp, and 5-HTP levels in the colon, feces, and serum; Balance excitatory and inhibitory neurotransmitter levels by restoring maternal VPA-induced decrease in GABA and Ach levels, and increase in Glu level and Glu/GABA in serum, the medial PFC or cerebellum of rats; Enhance oxytocin synthesis in the hypothalamus; Reduce the 5-HT associated *Turicibacter* in the gut; Increase butyric acid levels in the cecum contents	Kong et al. (2021)
*B. longum* BB536	Young Sprague Dawley male rats, oral gavage of 250 mg/kg propionic acid dissolved in distilled water for 3 days	2 × 10^9^ CFU per 25 mg dissolved in a volume of 1 mL of sterile PBS, 0.5 mL daily by oral gavage for 22 days	Improve the social behavior impairment; Decrease the harmful *C. perfringens* and increase *Clostridium* cluster IV; Normalize the PPA-induced increase in *Bdnf* transcript levels in the hippocampus	Abuaish et al. (2021)
*L. plantarum* STIII	Offspring of pregnant ICR mice administered with triclosan dissolved in fresh corn oil at concentration of 50 mg/mL, intragastric gavage from the 7^th^ day of pregnancy until the 21st day of weaning at a dose of 50 mg/kg	5 × 10^8^ CFU/g dissolved in PBS, 0.8 mL daily by intragastric gavage at the age of 7 weeks for 2 weeks	Ameliorate the social deficits, the self-grooming and freezing times; Increase the beneficial *Lachnospiraceae* abundance and decrease *Alistipes* abundance	Guo et al. (2022)
*L. paracaseii* LPC-37	Male Wister albino rats treated with 250 mg PPA/kg BW/day for 3 days	5 × 10^9^ CFU dissolved in 1 mL of sterile PBS, 0.2 mL daily by oral gavage for 27 days before PPA exposure	Reverse PPA-induced decrease in α-MSH levels, neurotensin, and β-endorphin	Alghamdi et al. (2022)
*B. infantis*, *B. breve*, *L. acidophilus*, L. *bulgaricus*, *L. casei*, *L. rhamnosus*, and *S. thermophiles*	Male Wister albino rats treated with 250 mg PPA/kg BW/day for 3 days	1 × 10^9^ CFU/g dissolved in PBS, 0.2 g/kg BW daily by oral gavage for 27 days before PPA exposure	Reverse PPA-induced decrease in α-MSH levels, neurotensin, and β-endorphin	Alghamdi et al. (2022)
Four *Lactobacillus* spp. and *Bifidobacterium* spp.	Adult Wistar rats received broad-spectrum antibiotics mixture for 4 weeks at age of 10 weeks old	Daily oral gavage for 2 weeks	Improve the social behavior; restore antibiotics-induced decrease in SCFAs	Mintál et al. (2022)
Four *Lactobacillus* spp. and *Bifidobacterium* spp.	Male offspring of pregnant Wistar rat intraperitoneal injection of 500 mg/BW kg VPA on the 12.5th day of gestion	Daily oral gavage for 2 weeks	Improve the social behavior	Mintál et al. (2022)
*S. thermophilus* BT01, *B. breve* BB02, *B. animalis* subsp. *lactis* BL03, *B. animalis* subsp. *lactis* BL04, *L. acidophilus* BA05, *L. plantarum* BP06, *L. paracasei* BP07, *L. helveticus* BD08.	Male offspring of pregnant Wistar rat intraperitoneal injection of 500 mg/BW kg VPA on the embryonic day 12.5	2.25 × 10^10^ CFU/day probiotic was administered *via* orogastric gavage for 42 days	Improve the sociability, social interaction, anxiety-liked behavior, and behavioral despair; Significantly reverse the VPA-induced increase in serum IL-6 and decrease in serum IL-10; Restore the Bacteroidetes/Firmicutes ratio decreased by prenatal VPA exposure	Adıgüzel et al. (2022)
*Lacticaseibacillus rhamnosus* HA-114	Male juvenile BTBR T^+^ Itpr3^tf^/J mouse	1 × 10^9^ CFU/ mL probiotic reconstituted in drinking water for 4 weeks	Reduce behavior deficits in social novelty preference; Increase microbial richness and phylogenetic diversity; increase the potential anti-inflammatory (*Anaeroplasma*, *Christensenellaceae*) and butyrate-producing taxa (*Acetatifactor*, *Lachnospiraceae*, and *Butyricicoccus*); Elevate levels of 5-aminovaleric acid and choline in serum and the PFC, respectively	Pochakom et al. (2022)

Second, probiotics can modulate neuroactive compounds to attenuate ASD symptoms. Accumulating evidence has demonstrated that genetic and environmental risk factors converge to disturb the balance between glutamate (Glu)-mediated excitatory and γ-GABA-mediated inhibitory neurotransmission autism (Nelson and Valakh, 2015; Borisova, 2018). Probiotics can influence neurotransmitters such as γ-GABA, Glu, and 5-HT (Ng et al., 2018; Israelyan and Margolis, 2019). Tabouy et al. (2018) revealed that *L*. *reuteri* treatment decreased repetitive behaviors and increased GABA receptor gene expression (GABRA1, GABRA1, and GABRB1) and protein levels (GABRA1) in the hippocampus and the PFC of Shank3 mutant mice (a model of ASD). Moreover, treatment with *Lactobacillus* was shown to regulate emotional behavior and central GABA receptor expression *via* the vagus nerve (Bravo et al., 2011), which communicates connecting the brain and the gut, in a mouse. Probiotics that stimulate inhibitory neurotransmission (for example, by increasing GABA levels) may help restore the excitatory/inhibitory balance and recover the decreased social interaction associated with ASD (El-Ansary et al., 2018). In addition, daily *L*. *helveticus* CCFM1076 intake alleviates autistic-related features by regulating 5-HT anabolism and catabolism, balancing excitatory and inhibitory neurotransmitter release (as indicated by increased GABA in PFC and decreased Glu in serum, and PFC) in both the peripheral and CNS, and increasing oxytocin synthesis in the hypothalamus (Kong et al., 2021). 5-HT is produced in the gut and plays a central role in gut–brain connection (Owens and Nemeroff, 1994). Previously, 5-HT levels have been significantly correlated with GABA, Glu, and oxytocin, suggesting a vital role of 5-HT in the neuroendocrine network. Moreover, a single dose of oxytocin has been shown to regulate the 5-HT energy system, reduce anxiety (Neumann and Slattery, 2016), and help alleviate social dysfunction (Lawson et al., 2016). Another neuropsychiatric disease involves the altered neurotransmitter Glu (Shimmura et al., 2011). ACh is involved in learning and memory, attention, cognition, social interactions, and stereotypical behaviors (Avale et al., 2011; Karvat and Kimchi, 2014). In addition, *L. reuteri* treatment raised oxytocin levels in the brain, which improved behavioral aspects of brain function by stimulating the vagus nerve (Sgritta et al., 2019). Another study found that *L*. *reuteri* ingestion restored maternal high-fat diet-induced social deficits, oxytocin levels, and ventral tegmental area plasticity in offspring (Buffington et al., 2016). Furthermore, *L. reuteri* has been repeatedly shown to improve oxytocin-dependent behavior in several ASD mice models (Sgritta et al., 2019). Brain-derived neurotrophic factor (BDNF) is a neurotrophic factor that promotes the development and survival of cholinergic, dopaminergic, and serotonergic neurons in their mature and growing stages (Croen et al., 2008). Working memory, hippocampal learning, and brain plasticity are all influenced by BDNF (Leung and Thuret, 2015). In addition, BDNF impacts GABA inhibitory interneurons, ultimately causing cognitive deficits (Maqsood and Stone, 2016). One previous study reported that daily *Lactobacillus* strains supplementation reversed autistic deficits and decreased BDNF levels in serum and acetylcholinesterase (AChE) and 5-HT in the brain of the VPA-induced prenatal model of autism (Sunand et al., 2020). Acetylcholine (Ach), hydrolyzed by AChE in the synaptic cleft (Croen et al., 2008), is involved in learning and memory, attention, cognition, social interactions, and stereotypical behaviors (Karvat and Kimchi, 2014). In a recent report, both the pure and mixed probiotics had beneficial effects against PPA-induced neurotoxicity shown by increased levels of alpha-melanocyte-stimulating hormone (α-MSH) levels, neurotensin, and β-endorphin in ASD of rodent model (Alghamdi et al., 2022). A remarkable decrease in α-MSH in different brain regions has been involved in the pathogenesis of social isolation (Theoharides and Doyle, 2008); in fact, re-socialization fully recovered α-MSH immunoreactivity attenuating anxiety-and depression-like behaviors (Tejeda et al., 2012). Neurotensin may act on the CNS as atypical neuroleptics (Petrie et al., 2005). β-endorphin, endogenous opioid peptides, may alter social behavior and result in autistic-like features. A probiotic mixture was shown to attenuate both the antibiotics and VPA-induced autistic behavioral symptoms (Mintál et al., 2022). In the BTBR mouse model of autism, probiotic *L. rhamnosus* administration decreased behavioral abnormalities in social novelty preference and increased 5-aminovaleric acid and choline levels in serum and the PFC, respectively (Pochakom et al., 2022). The excitatory/inhibitory imbalance previously linked to the pathophysiology of ASD is attenuated by 5-aminovaleric acid, a GABA receptor agonist, of which persons with ASD have remarkably lower levels than non-ASD ones (Sharon et al., 2019). The social and behavioral impairments observed in ASD have been connected to cholinergic pathways through choline metabolism (Lam et al., 2006). Choline supplementation during pregnancy and blocking Ach the breakdown both helped BTBR mice with social and repetitive/restricted behavior deficiencies (Eissa et al., 2020).

The reduction of gut inflammation (improved immune functions) may be another benefit of probiotic application for ASD. Several GI illnesses, including irritable bowel syndrome and inflammatory bowel disease, have been associated with increased mucosal inflammation (Ng et al., 2018). Children with ASD have been found to have greater levels of gut immune inflammation, which is linked to gut dysbiosis, as well as GI complaints (Hughes et al., 2018). In fact, 4 months of probiotic supplementation in children with ASD aged 2–9 years restored many of the abnormalities in their GI microbiota and reduced their intestinal inflammation (Tomova et al., 2015). Probiotics have been shown to reduce gut inflammation through numerous mechanisms including lowering gut barrier permeability, decreasing inflammatory cytokines, and other immunomodulatory effects. In pregnant female mice, maternal immune activation (MIA) results in impaired intestinal barrier integrity and symptoms like autism in the offspring, which are related to microbiome dysbiosis (Hsiao et al., 2013). After *Bacteroidetes fragilis* treatment, the repetitive behaviors were attenuated and intestinal permeability was restored, and the gut microbiota imbalance partially improved in the offspring (Hsiao et al., 2013). The probiotic mixture VSL#3 significantly improved sociability, social interaction, anxiety-liked behavior, and behavioral despair, while reversing the increase in serum IL-6 and decrease in serum IL-10 induced by prenatal VPA exposure (Adıgüzel et al., 2022). Moreover, daily *Lactobacillus* strain supplementation supports gut–brain axis in the VPA-induced prenatal model of autism by reversing autistic deficits and improving immune functions (Sunand et al., 2020). In their study, treatment with *Lactobacillus* strains decreased TNF-α levels in serum and IL-6 in the brain. TNF-and IL-1 attach to the brain’s endothelial cells to trigger immunological responses in the brain (de Theije et al., 2011). In addition, reduced IL-6 levels have been shown to enhance GABAergic interneuron activity, which in turn increases GAD65/67 levels, preventing the loss of parvalbumin-positive (PV^+^) neurons and GABA levels (Basta-Kaim et al., 2015).

## 5. Conclusion and future directions

In this review, we first showed the interrelationship between GI abnormality, gut microbiota dysbiosis, and ASD severity. Then, we presented how gut microbiota dysbiosis contributes to gut–brain axis dysfunction in patients with ASD. Finally, we indicated how probiotics affect the gut microbiota, leading to improvements in GI abnormalities and other behaviors by regulating the gut–brain axis.

Despite the encouraging preclinical and clinical results of probiotics supplementation, most accessible clinical studies had small sample sizes, most being single-center trials that enrolled only 20–30 children, and may use qualitative, self-reported questionnaires and surveys to measure treatment response in open-label trials, which might introduce bias. Due to the communication deficits that are common in children with ASD, the parents may also encounter several challenges while analyzing these aspects. The use of clinician ratings, more randomized, controlled research, and bigger study populations may produce more reliable findings. The long-term effects of probiotics in patients with ASD after cessation have not been investigated. Thus, it is necessary to prove the elution stage of probiotic administration in the future. Moreover, the lack of an established probiotic protocol results in a variety of probiotic strains, concentrations, and treatment times. Interestingly, probiotics were most useful when using certain strains and conditions (McFarland et al., 2018). Future research should consider using a standardized intervention plan. Mechanistic studies utilizing “multi-omics” may be used in the future. Recent technological advancements in the area of metabolomics have vastly improved the sensitivity and accuracy with which metabolites can be detected and characterized (Du et al., 2017; Wang et al., 2019). To progress the discipline even further, bigger studies using a defined intervention protocol and the development of metabolomics are also required. In summary, patients with neurodevelopmental disorders, such as ASD, may benefit from a well-chosen mix of probiotics as a potential non-invasive therapy.

## Author contributions

PF and SZ co-wrote the manuscript. YZ revised the manuscript. EL supervised the manuscript. All authors contributed to the article and approved the submitted version.

## Funding

This study was supported by the Natural Science Foundation of Henan Province (No. 212300410399) and the Zhengzhou Major Collaborative Innovation Project (No. 18XTZX12003).

## Conflict of interest

The authors declare that the research was conducted in the absence of any commercial or financial relationships that could be construed as a potential conflict of interest.

## Publisher’s note

All claims expressed in this article are solely those of the authors and do not necessarily represent those of their affiliated organizations, or those of the publisher, the editors and the reviewers. Any product that may be evaluated in this article, or claim that may be made by its manufacturer, is not guaranteed or endorsed by the publisher.
